# Digital literacies, social media, and undergraduate learning: what do students think they need to know?

**DOI:** 10.1186/s41239-023-00398-2

**Published:** 2023-05-19

**Authors:** Erika E. Smith, Hannah Storrs

**Affiliations:** 1 Academic Development Centre, Mount Royal University, Calgary, Canada; 2 Mount Royal University, Calgary, Canada

**Keywords:** Undergraduate students, Digital literacy, Digital literacies, Social media, Higher education

## Abstract

This research addresses an identified need to further understand digital literacies (DL) and whether undergraduate students view DL as being important in their lives and in their learning. Using a cross-sectional survey sent to a stratified random sample of 2500 undergraduates representative of the overall student population at a medium-sized Canadian undergraduate university (survey response rate of 19.8%, *N* = 496), we explored the relationships between social media and digital literacies, particularly in different disciplinary contexts. We also explored the ways in which students report using social media in their university learning, showing that students value social media for collaboration, discussion, information finding and sharing, and practise activities related to their learning. Additionally, we examined the importance students place on DL, and how they perceive and rate their own abilities with digital literacies across three domains: procedural and technical, cognitive, and sociocultural. Findings illustrate an observable gap between the high importance that students place on digital literacies (including DL for social media) in their learning and their lives and the lack of coverage students reported receiving about these topics in their undergraduate education. Based on the study’s findings, we discuss the specific ways that those in the higher education community can address this gap by engaging with and fostering development of digital literacies within specific disciplinary and professional contexts, and in interdisciplinary or transdisciplinary learning settings across the curriculum.

## Introduction

Over the past decade, there has been a noticeable increase in the use of social media technologies in post-secondary teaching and learning, and this growth only continued during the COVID-19 pandemic (Katz & Nandi, 2021; Khan et al., 2021). As social media continues to be an ever-present part of students’ lives and their learning, it is important to understand their experiences with and uses of social media and the related digital literacies required to engage with these effectively and meaningfully.

While prior research has explored the social media technologies that post-secondary institutions and faculty use, there is a need for further research exploring students’ perceptions and uses of social media to aid their learning (Hamid et al., 2015). Through their in-depth review and analysis, Littlejohn et. al. (2012) also concluded “that institutions need to place greater value on ‘literacies of the digital’, and better prepare their students and their own organizational processes to thrive in an age of digital knowledge practices” (p. 547). Additionally, EDUCAUSE’s report on digital literacy in higher education highlighted building “literacy across disciplines” (Alexander et al., 2016, p. 6) as an area requiring further attention. Based on such needs identified in prior research, we explored undergraduate students’ use of social media in their learning, how they perceive and rate their own DL abilities, the importance they place on digital literacies including those related to social media, and the extent to which these topics were covered in their undergraduate education. While the setting of our survey was a Canadian university, our research echoes and expands upon broader contemporary work on digital literacies and social media in higher education across North America and the Western world. Our inquiry therefore emerged from the needs identified in the literature, as well as calls to action from the educational technology community (e.g., EDUCAUSE), and key findings of a prior study (Smith, 2017), demonstrating that further research should explore relationships between social media and digital literacies and identify opportunities for developing DL in undergraduate learning, particularly in different disciplinary contexts.

## Literature review

Although digital technologies, and social media in particular, have become essentially ubiquitous today, it is problematic to conflate this prevalence with effective use of these technologies. While there are many definitions of what constitutes DL, as described in greater detail below, we view students’ effective use of digital technologies as requiring competences that integrate procedural and technical, cognitive, and sociocultural abilities that are applied in different contexts. As Cohen and Hewitt (2019) describe, “there can be a false assumption that students who grew up with technology are successful using it across contexts and in multiple areas—including in their college classes” (para. 4), pointing out that while research shows a majority of faculty believe their students are well-prepared to use technologies, students themselves express discomfort in applying these tools in their learning and in different academic settings. There is an opportunity to debunk such false assumptions in tandem with fostering faculty educators’ own digital competencies and literacies through ongoing education and professional development initiatives, where integrating evidence-informed understandings of students’ needs, abilities, and skills are invaluable.

Inaccurate assumptions about students’ default skills and abilities are often reinforced by ideas of students as *digital natives*, a term used by Prensky (2001) to describe a generation of learners who have grown-up immersed in technology and are presumed to be intrinsically adept users as a result. While simplistic constructs of digital natives have been largely debunked in the educational technology literature, these assumptions and stereotypes are still quite pervasive today, despite the wide body of research to the contrary (Smith et al., 2020). Araújo-Vila et. al.’s (2020) recent study shows that students often do not possess the abilities that they need to be successful with digital technologies in their learning, and face challenges applying the skills they do have at the level expected, making curricular initiatives that explicitly build these literacies imperative. By more deeply understanding students’ perceptions of and experiences with social media technologies and related literacies, especially those most critical to their undergraduate learning, we can ascertain where there are gaps to be filled and bolster curricular approaches to meet these needs.

To address the needs identified, our research aims to explore the following overarching questions:

i. What digital literacies do undergraduate learners from different disciplines view as important when using social media in their learning?
ii. Is there a relationship between the digital literacies that learners perceive to be important and their ability to apply these digital literacies to support learning in their discipline?

These questions guided our inquiry and provided the focus for our survey.

### Key definitions

Herrington et. al. (2010) described *social media* as cognitive tools and emerging technologies that “allow the creation of collaborative, shared knowledge…and the development of participatory cultures” (p. 9). Following from prior research and the survey instrument employed by Smith (2016, 2017), the following definition was used within the survey instrument for this study: social media include applications and websites that allow users to create and share content. Social media also enables users to connect via web technologies or to participate in social networks.

The term *digital literacy* has expanded considerably in both its scope and usage (Smith et al., 2018, 2020) since Gilster (1997) popularised it several decades ago. However, even in Gilster’s original definition, there has been an emphasis on the need to enact a broader meaning of literacy in digital contexts beyond simple technological aptitudes, a need for literacy that involved “mastering ideas, not keystrokes” (1997, p. 15). Digital competences and digital literacies are intertwined since, as Martin (2006) explains, digital competence is the foundational stage (or level of engagement) of digital literacy. As a key foundation, then, digital competence is often framed as being underpinned by digital literacy (Martin & Grudziecki, 2006; Spante et al., 2018). In the decades following Gilster’s work, more detailed conceptions of a plurality of *digital literacies* have been articulated (Alexander et al., 2017), often in ways that relate to an array of other literacies involving digital technologies. These related literacies include abilities for effectively finding, evaluating, and using information known as *information literacies* (American Library Association, n.d.). Additionally, digital literacies are often related to or encompassed within concomitant concepts of *new literacies* required in the context of rapidly evolving communication technologies (Coiro et al., 2014; O’Byrne, 2010), *multiliteracies* for “the increasing range of text forms that are associated with information and multimedia technologies” including multimodalities (Mills, 2010, p. xiii), *media literacies* “that are necessary for full participation in our media-saturated, information-rich society” (Hobbs, 2010, p. vii), and *transliteracies* needed for “a fluidity of movement across a range of technologies, media and contexts” (Sukovic, 2016, para. 2). Our research focuses on *digital literacies* (DL) and we use this term plurally to acknowledge the multifaceted and interconnected nature of these concepts (Spante et al., 2018).

Given the plurality at the heart of digital literacies, it is not surprising that there are many different (and, at times, diverging) definitions of DL. As Martzoukou et. al. (2020) describe, “the definition, terminology, ownership and responsibilities created within this domain are contested, with a plethora of debates and different opinions in respect to what individuals should master and accomplish in order to become sufficiently ‘digital’” (p. 1414). Considering the evolving nature of both the digital landscape and the concepts at hand, we view the development of digital literacies as being an ongoing, lifelong process, and agree with Martin (2006) who describes DL as follows:

Digital literacy is an ongoing and dynamic process—it is not a threshold which, once achieved, guarantees familiarity with the digital for ever after…. Maintenance of digital literacy is therefore ongoing; it is necessary to return again and again to the well of digital competence (whose contents are themselves changing as technology evolves) to acquire the competence needed to succeed in the life-situation, whether it be learning, work or leisure. (p. 156)

In addition to seeing DL as a cyclical, lifelong process, since the term digital literacies is typically used today in a plural form, Spante et. al. (2018) note that its use “acknowledges new and diverse social practices” (p. 7), as literacies can be contextualized in relation to social institutions and power relations.

In our research, and specifically in our survey instrument, we referred to the American Library Association Digital Literacy Task Force’s well-recognized definition of digital literacy as “the ability to use information and communication technologies to find, evaluate, create, and communicate information, requiring both cognitive and technical skills” (American Library Association, 2012, para. 1). Additionally, according to Jisc, a leading digital, data, and technology agency in the United Kingdom that focuses on tertiary education, research, and innovation, digital literacy should be understood as a developmental process that provides the “capabilities which fit an individual for living, learning and working in a digital society” (Jisc, 2014, Developing Digital Literacies section, para. 1). These definitions informed our work.

## Conceptual and theoretical framing

In grappling with the multiplicity of literacies connected with the use of digital technologies, Ng (2012) articulated the intersection of several DL dimensions as a conceptual framework, stating that “within educational contexts, digital literacy is a broader term that embraces technical, cognitive and social-emotional perspectives of learning with digital technologies, both online and offline” (p. 1066). While we found value in engaging with and building upon earlier conceptions of DL, such as those within Ng’s (2012) oft-cited framework, we also heeded Alexander et. al.’s (2017) recommendations to expand existing articulations of DL in ways that respond to digital divides and power dynamics that are sociocultural in nature. We recognized a need to integrate the strong foundation of educational research on sociocultural learning theories and practices in a digital age, including those relevant to critical and constructivist aims and approaches.

A conceptual framework that reflected our contemporary context and that embedded changes that incorporated, among other things, important sociocultural aspects of digital literacies was warranted. Building from recognized definitions, as well as an analysis of key concepts and a critical review of the literature on digital native discourse and DL, we therefore used three interconnected domains of digital literacies to conceptually frame our research:

1. Procedural and technical: the operational, procedural, or technical skills required to functionally utilise technologies effectively and productively.
2. Sociocultural: the meaningful ways in which technologies are shaped by and reflect sociocultural contexts within which they are developed and employed; and,
3. Cognitive: the need to process and relate information regarding cognitive aspects of technology use, for instance, by forming schemas for information retrieval and making metacognitive connections (Smith et al., 2020, p. 4).

These key constructs informed and guided our research approach, including items within the survey.

As a theoretical grounding for our study, we use a social constructivist approach, within which learning is understood as occurring when people construct meaning through their broader social interactions, experiences, and contexts (Driscoll, 2005). The premises of social constructivism, in particular interactional elements, also align with the affordances of social media (Dron & Anderson, 2014). As Martin (2006) describes, within constructivist and student-centred learning models, students’ development of DL and mastery of digital tools becomes a key factor in their success in the learning environment. Creswell and Creswell (2023) note that social constructivist research frameworks place emphasis on multiple meanings, and as such, this approach well grounds our work because undergraduate students’ views, meanings, and perspectives are the focus of our study.

## Research design and methodology

We employed a survey methodology using a cross-sectional design that included questions regarding students’ perceptions and self-reported uses categorically and on a continuum, and which enabled macro-level analysis as well as comparison of different groups (Cohen et al., 2018; Creswell & Creswell, 2023). This research paper focuses on the quantitative survey items related to digital literacies and social media in undergraduate learning, including whether differences may exist according to discipline or year of study. Approval for this study was obtained from the institutional Research Ethics Board, and in recognition of their time, participants could choose to enter a draw for a $40 gift card following the survey.

### Setting, sample, and data collection

Participants were recruited from a public, undergraduate-only university in Western Canada. This medium-sized university emphasizes personalized learning opportunities through smaller class sizes and lower student-to-instructor ratios, with a focus on face-to-face course delivery. This post-secondary institution integrates a General Education program across the curriculum as a part of its degree requirements and had 10,112 full load equivalent students enrolled at the time of the survey.

The survey instrument was primarily composed of close-ended Likert-type questions to capture student perceptions and ratings, as well as several open-ended questions to enhance validity. Drawing on her prior experience creating a survey of undergraduates on similar topics, the first author designed and constructed the instrument. The second author, who was an undergraduate Research Assistant in the student population at the time (ineligible and excluded from completing the survey), then tested the survey and provided detailed feedback that helped refine the instrument prior to its circulation. As a part of the validation process, researchers consulted the literature and also sought feedback on the survey instrument and on overall planning considerations for the survey administration from experts in the university’s Office of Institutional Analysis and Planning, and from a Professor Emerita and Librarian with expertise and experience in the subject area and with the student population. Reliability tests showed consistency, with Cronbach’s alpha above 0.8 for the Likert-type scale items reported below.

To ensure our random sample was representative of the student population, we received permission to send our electronic survey invitation via the university’s Office of Institutional Research and Planning distribution system. Survey invitations were sent to a stratified random sample of 2500 students representative of the overall population in terms of gender identification, year of study (i.e., across all years), and programs (i.e., degree, diploma, etc.). In total, there were 496 survey participants, for a response rate of 19.8% following data cleaning to remove missing or incomplete responses.

### Data analysis

Survey items were analysed using descriptive and inferential statistical procedures within SPSS software. Likert-type scales ranged from 5 (e.g., *strongly agree*, *a great deal, extremely competent*, etc*.*) to 1 (*strongly disagree*, *not at all, not at all competent*), as well as an option to indicate “I don’t know.” As the treatment of Likert-type scales has been a topic of some debate in the literature (see, for example, Jamieson, 2004), in analysing this data we conducted both a parametric and a corresponding non-parametric test and reported the most conservative *p* value (López et al., 2015; Polit, 2009). Effect sizes for all inferential tests, using Cramer’s V for chi-squared tests, Cohen’s *d* for t-tests, and partial eta-squared for one-way ANOVA tests, are also reported. Post-hoc tests (Bonferonni, Tamhane’s T2, and Scheffe) were conducted to further analyse differences between groups.

## Results

Undergraduate students (*N* = 496) from across disciplines participated in the survey: Humanities (including the Arts) and Social Sciences (*N* = 291, 59.1%), Health Sciences (*N* = 105, 21.3%), Sciences (*N* = 72, 14.6%), and Open Programs (with no specific discipline or profession) (*N* = 24, 4.9%). Students participated across all years of study, from first year (*N* = 69, 13.9%), second year (*N* = 158, 31.9%), third year (*N* = 143, 28.8%), and fourth year (*N* = 126, 25.4%). While this was a relatively normal distribution, a lower number of first year students responded to the survey, possibly due to survey fatigue since another university-wide survey ran prior to this one. Most respondents were between 20 and 25 years of age (*N* = 368, 77.4%), and in terms of gender identification, a small proportion of participants identified themselves as non-binary (*N* = 2, 0.4%), with a greater proportion of participants identifying as female (*N* = 354, 71.7%) as compared to male (*N* = 138, 27.9%).

### Undergraduate use of social media

Confirming results of prior research (Smith, 2016, 2017), a majority of students reported using social media in their university learning (*N* = 372, 75.2%), as well as in their everyday life (*N* = 459, 92.9%). For social media use in their everyday life, chi-square tests showed no statistically significant differences between credential type (i.e., earning a degree, diploma, or certificate), but did show significant differences between disciplinary categories (X^2^ (3, *N* = 490) = 19.90, Cramer’s *V* = 0.202, *p* < 0.001), year of study (X^2^ (3, *N* = 494) = 16.77, Cramer’s *V* = 0.184, *p* < 0.001), and gender (X^2^ (2, *N* = 492) = 14.35, Cramer’s *V* = 0.171, *p* = 0.002). In terms of disciplinary differences, more in the Health Sciences (*N* = 101, 96.2%) and the Humanities and Social Sciences (*N* = 274, 94.8%) indicated using social media in their everyday life than those in in the Sciences (*N* = 58, 80.6%). Additionally, a greater number of students in first year (*N* = 64, 94.1%), second year (*N* = 155, 98.1%), and third year (*N* = 133, 93.0%) indicated using social media in their everyday life than those in fourth year (*N* = 107, 85.6%). A significantly higher percentage of students identifying as female (*N* = 338, 95.8%) as compared to male (*N* = 118, 86.1%) indicated that they use social media in their everyday life. However, differing from the responses for use of social media in everyday life, in their own university learning, chi-square tests showed no statistically significant differences for social media use according to disciplinary category, year of study, credential type, or gender, indicating that social media use for university learning is relatively constant across these groups.

Students who reported using social media in their learning were asked to rank the following activities in terms of their value. Overall, undergraduates placed the highest value on information searching, collaboration, information sharing, help seeking, discussion, and logistical functions (e.g., organizing materials, schedule management), as shown in Table 1.

**Table 1:** Value of social media activities for learning

In your opinion, are the following social media activities valuable* for your university learning?	Mean (*SD*)	Percent (*N*)				
		Extremely valuable	Very valuable	Somewhat valuable	Not so valuable	Not at all valuable
Building relationships with peers (e.g., Facebook, LinkedIn)	3.61 (1.00)	19.5% (71)	36.7% (134)	32.1% (117)	8.5% (31)	3.3% (12)
Building relationships with instructors (e.g., Facebook, LinkedIn)	2.91 (1.18)	12.1% (44)	18.1% (66)	29.9% (109)	28.8% (105)	11.2% (41)
Looking for help from others online	4.04 (0.92)	35.4% (128)	40.9% (148)	17.4% (63)	5.2% (19)	1.1% (4)
Searching for information online	4.63 (0.73)	74.3% (269)	18.0% (65)	5.2% (19)	1.7% (6)	0.8% (3)
Sharing information online with others (e.g., (re)posting links to websites, articles)	4.04 (0.94)	38.1% (139)	34.8% (127)	21.4% (78)	4.7% (17)	1.1% (4)
Commenting on online information	3.14 (1.17)	15.9% (58)	19.8% (72)	34.3% (125)	22.0% (80)	8.0% (29)
Creating media to share online (e.g., pictures, videos, music)	3.41 (1.23)	23.3% (85)	26.3% (96)	26.6% (97)	15.9% (58)	7.9% (29)
Collaborating with others to create things online (e.g., Google Docs)	4.66 (0.65)	74.5% (272)	18.9% (69)	5.2% (19)	1.1% (4)	0.3% (1)
Discussing ideas with others online (e.g., messaging, forums)	4.17 (0.93)	45.2% (165)	33.7% (123)	15.1% (55)	5.2% (19)	0.8% (3)
Managing your own academic schedule	4.12 (1.08)	49.9% (182)	23.8% (87)	17.5% (64)	5.8% (21)	3.0% (11)
Practising what you are learning (e.g., digital simulations)	3.81 (1.11)	34.8% (127)	26.8% (98)	25.5% (93)	10.1% (37)	2.7% (10)
Organizing learning materials (e.g., Google Drive)	4.55 (0.72)	64.1% (234)	29.0% (106)	5.2% (19)	0.5% (2)	1.1% (4)

*Scale: (5) extremely valuable, (4) very valuable, (3) somewhat valuable, (2) not so valuable, and (1) not at all valuable

The top types of social media that students overall reported using most in their everyday life were in the categories of social networking (e.g., Facebook, *N* = 376, 75.8%), image sharing (e.g., Flickr, Instagram, Pinterest, *N* = 363, 73.2%), Google Apps (e.g., Google Docs, *N* = 343, 69.2%), instant messaging or VOIP (e.g., Skype, Google Hangouts, WhatsApp, Snapchat, Discord, *N* = 334, 67.3%), video sharing (e.g., YouTube, *N* = 332, 66.9%), and file sharing (e.g., Dropbox, Google Drive, BitTorrent, *N* = 232, 46.8%). Conversely, the top types of social media that students indicated using most in their university learning were in the categories of Google Apps (e.g., Google Docs, *N* = 336, 67.7%; as a note, this university has an institutional version of Google Apps for Education), file sharing (e.g., Dropbox, Google Drive, BitTorrent, *N* = 271, 54.6%), video sharing (e.g., YouTube, *N* = 222, 44.8%), instant messaging or VOIP (e.g., Skype, Google Hangouts, WhatsApp, Snapchat, Discord, *N* = 159, 32.1%), wikis (e.g., Wikipedia, *N* = 194, 39.1%), and social networking (e.g., Facebook, *N* = 149, 30.0%).

### Digital literacies: students’ perceived needs, learning coverage, and abilities

When asked about the need for digital literacies, as shown in Table 2, a majority of students agreed or strongly agreed that they need digital literacies for a range of activities in their learning, in their disciplinary or professional context, and in their daily lives. Conversely, few students reported learning a lot or a great deal about these same digital literacies. As Table 3 illustrates, a majority of students reported learning a moderate amount, a little, or not at all about these digital literacies in their undergraduate education. Notably, while over three-quarters of students agreed or strongly agreed that they need digital literacies to effectively use social media, most reported not learning much about this in their undergraduate education.

**Table 2:** Need for digital literacies

Please indicate your level of agreement* with the following statements:	Mean (*SD*)	Percent (*N*)					
		SA	A	N	D	SD	I don’t know
I need digital literacies							
For functions in my everyday life	4.00 (1.00)	34.0% (161)	44.6% (211)	12.3% (58)	6.3% (30)	2.3% (11)	0.4% (2)
To be an active citizen in my community	3.78 (1.01)	24.1% (114)	44.2% (209)	20.1% (95)	9.3% (44)	1.7% (8)	0.6% (3)
To effectively use social media	4.07 (1.02)	36.4% (172)	46.0% (217)	10.2% (48)	4.2% (20)	1.5% (7)	1.7% (8)
To be successful in my university learning	4.04 (1.05)	39.6% (187)	37.9% (179)	13.1% (62)	6.1% (29)	2.5% (12)	0.6% (3)
For my future profession or discipline	4.17 (1.02)	44.8% (212)	37.6% (178)	11.4% (54)	3.2% (15)	1.3% (6)	1.7% (8)

*Scale: (5) strongly agree, (4) agree, (3) neutral, (2) disagree, and (1) strongly disagree

**Table 3:** Amount learned about digital literacies

During your time at [this university], how much have you learned* about the following digital literacies?	Mean (*SD*)	Percent (*N*)					
		A great deal	A lot	A moderate amount	A little	Not at all	I don’t know
Digital literacies that I need							
For functions in my everyday life	2.83 (1.19)	10.0% (47)	14.8% (70)	39.8% (188)	20.6% (97)	12.9% (61)	1.9% (9)
To be an active citizen in my community	2.69 (1.26)	7.6% (36)	18.5% (87)	32.5% (153)	22.7% (107)	14.6% (69)	4.0% (19)
To effectively use social media	2.54 (1.36)	9.6% (45)	15.5% (73)	26.1% (123)	20.4% (96)	25.1% (118)	3.4% (16)
To be successful in my university learning	3.34 (1.26)	19.5% (92)	28.5% (134)	31.0% (146)	10.6% (50)	8.3% (39)	2.12% (10)
For my future profession or discipline	3.16 (1.34)	18.3% (86)	24.8% (117)	28.0% (132)	15.7% (74)	10.0% (47)	3.2% (15)

*Scale: (5) a great deal, (4) a lot, (3) a moderate amount, (2) a little, and (1) not at all

Even though they reported not learning much about digital literacies, including those needed for social media, in their formal undergraduate education, most students rated themselves as being very or extremely competent in their own skills and abilities to effectively use social media, as shown in Table 4. Interpretation of these findings, however, should also take into account that people (including students) generally tend to overrate their skills and abilities (Schlösser et al., 2013). There are some differences between the three DL domains, as students view themselves as being most competent in using social media technically and cognitively, providing somewhat lower ratings for their sociocultural skills and abilities.

**Table 4:** Self-rated abilities to use social media effectively

How would you rate your own skills and abilities to effectively use social media in the following areas?	Total *N* (*n*, %)*	Mean (*SD*)
Technical abilities (e.g., skills for operating social media settings, profiles, accounts, etc.)	471 (354, 75.2%)	4.01 (0.90)
Cognitive abilities (e.g., critical thinking, information, learning how to learn about social media technologies)	472 (361, 76.5%)	3.97 (0.88)
Sociocultural abilities (e.g., understanding social and cultural contexts of interactions on social media, etc.)	472 (335, 70.9%)	3.91 (0.97)

*Number and percentage of (4) very competent and (5) extremely competent responses

### Trends for digital literacies by discipline

To determine whether there were differences between disciplines for students’ agreement on the need for digital literacies, we conducted a one-way analysis of variance (ANOVA). The ANOVA showed significant differences between disciplinary groups in terms of needing digital literacies for their future profession or discipline (*F*(3,465) = 2.87, *p* = 0.036, η^2^ = 0.013), with the Bonferroni post-hoc test indicating that significant differences between students in the Sciences (*M* = 3.88, *SD* = 1.30) who provided much lower agreement compared to those in the Humanities and Social Sciences (*M* = 4.26, *SD* = 0.99): *p* = 0.036. Regarding students’ self-rated skills and abilities to effectively use social media, though post-hoc tests did not determine where particular disciplinary differences occurred, an ANOVA found significant differences between disciplinary groups regarding their sociocultural abilities: *F*(3,464) = 2.69, *p* = 0.046, η^2^ = 0.010.

We also conducted analyses to determine whether there were differences between disciplinary groups for how much they reported learning about digital literacies during their time at university. An ANOVA showed significant differences between disciplines regarding how much they learned about DL for being an active citizen in their community (*F*(3,464) = 4.01, *p* = 0.008, η^2^ = 0.002), for using social media (*F*(3,464) = 4.10, *p* = 0.007, η^2^ = 0.002), and for their future profession or discipline (*F*(3,464) = 2.95, *p* = 0.032, η^2^ = 0.005). In terms of learning about DL for being an active citizen in their community, Tamhane post-hoc tests illustrated that students in the Health Sciences (*M* = 2.44, *SD* = 1.22) reported learning significantly less than those in the Humanities and Social Sciences (*M* = 2.85, *SD* = 1.21), *p* = 0.031. Similarly, regarding the amount they learned about DL for using social media, Tamhane post-hoc tests illustrated that students in the Health Sciences (*M* = 2.26, *SD* = 1.22) again reported learning significantly less than students in the Humanities and Social Sciences (*M* = 2.71, *SD* = 1.40), *p* = 0.018. When asked about learning DL for their future profession or discipline, Tamhane post-hoc tests showed that students in the Humanities and Social Sciences (*M* = 3.25, *SD* = 1.33) also indicated learning more than their counterparts in an Open Program (*M* = 2.50, *SD* = 1.22), *p* = 0.047.

### Trends for digital literacies by gender and year of study

When examining responses according to year of study, ANOVA tests showed no significant differences between student responses on their agreement regarding the need for digital literacies, nor were there significant differences on students’ self-rated skills and abilities to effectively use social media. However, an ANOVA did show significant differences according to year of study for how much students reported learning about the digital literacies they need to function in their everyday life (*F*(3,468) = 2.68, *p* = 0.047, η^2^ = 0.019), to be an active citizen in their community (*F*(3,467) = 4.05, *p* = 0.007, η^2^ = 0.018), for using social media (*F*(3,467) = 3.18, *p* = 0.024, η^2^ = 0.009), to be successful in their university learning (*F*(3,467) = 4.62, *p* = 0.003, η^2^ = 0.007), and for their future profession or discipline (*F*(3,467) = 3.31, *p* = 0.020, η^2^ = 0.021). Tamhane post-hoc tests indicated that differences largely occurred between first year students who reported learning significantly less about digital literacies than those in higher years of study, showing differences between first and fourth year students in particular. There was one exception to this overall pattern, as second year students (*M* = 3.53, *SD* = 1.20) reported learning more than third years (*M* = 3.12, *SD* = 1.25) about the DLs needed to be successful in their university learning, *p* = 0.029.

While no significant differences according to gender were found regarding the amount students reporting learning about DL, or for students’ self-rated abilities to use social media effectively, a t-test examining how students rate the need for digital literacies found that, as compared to males (*M* = 3.89, *SD* = 1.14), female students (*M* = 4.14, *SD* = 0.93) provided significantly higher agreement that they need digital literacies to effectively use social media: *t*(467) = − 2.50, *d* = − 0.257, *p* = 0.013.

### Summary of results

Results of the survey confirm prior research by showing the majority of undergraduate students use social media in their university learning and in their everyday lives. While the results do not show differences between groups for social media use in their own university learning, they did show significant differences between groups in students’ use of social media in their everyday lives. For example, students in Health Sciences and the Humanities and Social Sciences reported higher use of social media in their everyday lives versus those in the Sciences, and students in the Humanities and Social Sciences also indicated higher agreement on the need for digital literacies for their future profession or discipline and generally reported learning more about digital literacies during their time at university. There were also differences for everyday use of social media between groups according to year of study, with lower use reported by fourth year students, and results also showed significant differences according to year of study for how much students reported learning about the digital literacies, with differences largely between those in early versus later years of study, and between first and fourth year students in particular. There were also differences for social media use in everyday life according to gender identification, with higher use indicated by females than males, and female students also provided significantly higher agreement that they need digital literacies to effectively use social media.

Results also highlight differences in the most common types of social media platforms students reported using in their everyday lives as compared to the ones they use in their university learning, suggesting that the affordances of social platforms play an important role. When using social media in their own learning, the types of activities students placed the highest value on (Table 1) were information searching, collaboration, information sharing, help seeking, discussion, and logistical functions (e.g., organizing materials, schedule management). While the majority of students agreed or strongly agreed that they need digital literacies to effectively use social media (Table 2), most reported not learning much about this in their undergraduate education (Table 3), and students provided lower ratings of their own efficacy with sociocultural (versus cognitive or technical) skills and abilities on social media (Table 4).

## Discussion

Survey findings show that the majority of undergraduate students report use of social media in their learning and in their lives. Students’ use of specific types of social media are relatively consistent with prior research in this area (Smith, 2017), particularly regarding students’ use of video sharing websites such as YouTube, often to (re)view subject-related content (Henderson et al., 2015). Students’ use of social networking platforms such as Facebook in learning has decreased from previous studies, which may in part be explained by the higher use of image sharing social platforms such as Instagram. As described in the *State of Social Media in Canada* reported just after our survey was conducted, Instagram use largely increased (up 22%) while Facebook use declined (down 11%) for those aged 18–24 (Gruzd & Mai, 2020), and this age group continues to be the dominant group (87%) on Instagram today (Mai & Gruzd, 2022).

### Students value interaction and collaboration on social media

Results of this survey also show that undergraduates rated social media as being very valuable for many activities related to their learning, and reinforce Henderson et. al.’s (2015) findings that student engagement with digital technologies generally occurs in two areas: student logistics and student learning. Similar to Henderson et al.’s findings, students in our study placed higher value on traditional “transmission” activities, such as searching for information, whereas they placed lower value on media creation activities. However, many of these activities also reflect self-regulated learning that is a core part of undergraduate education, and reinforce prior findings on students’ use of social media for help-seeking activities (Hayman et al., 2019).

Additionally, a notable finding is that students placed high value on social media for collaboration, discussion, sharing, and practise activities related to their learning, results that reinforce social constructivist aspirations of social media for learning beyond passive knowledge consumption, as articulated by Dron and Anderson (2014) among others. In these ways, the findings from this study reflect similar patterns to results of a prior survey (Smith, 2017) regarding the usefulness of social media activities, especially regarding low ratings for using social media to build relationships with instructors online: only 12.9% of students strongly agreed with this in the prior study, quite similar to the 12.1% of students indicating social media is extremely valuable for this purpose in the current study. In previous research, student interviews and survey responses illustrated the prominence of social media for student–student and student–content interactions, and less so for faculty–student interactions (Smith, 2016), a finding that appears to be congruent with our current research.

### Self-reported abilities with social media

Students rated themselves more highly in their technical and cognitive abilities, with somewhat lower ratings of their sociocultural DL, pointing to opportunities for developing university students’ sociocultural skills and abilities with social media in the curriculum. In general, students rated themselves as being very or extremely competent users of social media. To understand these findings, we can look to research that has established that higher education students tend to overestimate their skills and abilities related to digital and information literacies (Jankowski et al., 2018; Mahmood, 2016; Morgan et al., 2022). These high self-ratings may be explained by “the tendency for people to overrate their skill, expertise, and performance. People provide overinflated views of themselves in a variety of settings” (Schlösser et al., 2013, p. 86), a phenomenon often referred to as the Dunning–Kruger effect. Similarly, these findings may reflect self-enhancement bias, a tendency for people to give more favourable self-views than are objectively warranted (Weiner & Guenther, 2020, para. 1). Nonetheless, understanding student perceptions and self-rated abilities is important for many reasons, including their representation of the beliefs that often inform actions and motivations. As Mahmood (2016) describes, self-perceptions studies are one of the most popular ways of evaluating student skills since “[s]elf-efficacy, based on self-perceptions regarding particular behaviors, influences human functioning and is considered important for lifelong learning” (p. 200). Potential disconnects between students’ perceptions and application of their digital literacies in a variety of contexts is an area where undergraduate education can look to beneficially hone this self-efficacy.

### Gaps between needs for and coverage of digital literacies

While students highly agreed that they need digital literacies, they reported being taught little about these digital literacies (including DL needed to effectively use social media), illustrating observable gaps between students’ needs and their experiences in the undergraduate curriculum. While some of these gaps could potentially be explained by differences in discipline or may lessen somewhat as students progress through programs, consistent with the overall results shown in Table 3, students’ mean responses across all disciplinary groups and years of study still hovered at or below mid-scale, indicating they learned only a little or a moderate amount about digital literacies. These results are consistent with reports in research and practice identifying a need for greater coverage of digital literacies in the undergraduate curriculum. For example, a survey by the Canadian Digital Learning Research Association recently emphasized both faculty and student digital literacy as a challenge area (Johnson, 2022). Challenges for teaching DL in higher education have been further described by Cohen and Hewitt (2019) as follows:

Regardless, instruction in digital literacy acquisition is often inconsistent, both from campus to campus and even among students on the same campus. On top of that, many faculty do not integrate digital skills in the context of their subject or discipline because they are not comfortable with technology themselves, nor do they have the time in their courses to cover this area. (para. 6)

These inconsistencies in covering digital literacies within disciplinary contexts was reinforced in our findings. For instance, students within the Humanities and Social Sciences often reported greater coverage of these topics than students in other disciplines, such as those in the Health Sciences. While it is somewhat unsurprising that program areas in the Humanities and Social Sciences, including Arts, Business, and Communications (e.g., Journalism, Information Design, Public Relations, etc.), may integrate topics related to social media within their curriculum, areas within the Health Sciences, including Nursing and Midwifery, also typically have professional standards around social media use that are just as critical (Smith et al., 2020). When considering the disciplinary and professional needs for digital literacies across areas, as well as bridging students’ academic needs with those present in their everyday lives, addressing such DL gaps and inconsistencies becomes imperative.

Additionally, findings from the first author’s prior research (Smith, 2016, 2017) of undergraduate perceptions of social media in their learning found that students themselves expressed a need to further understand specifically why and how certain social media technologies should (or can) be used for learning in meaningful ways, and had underscored the importance of including digital literacies as a part of a comprehensive undergraduate education, particularly in ways that foster their abilities for integrating beneficial aspects of social media and mitigating the drawbacks. All of this points to a need to actively foster development of DL for students’ learning and for their everyday lives in ways that reflect digital citizen attributes (Blaj-Ward & Winter, 2019) as an iterative, cyclical, lifelong process.

Within the undergraduate setting, DL initiatives can therefore aim to strike a balance between building knowledge and skills within disciplinary coursework (Nelson et al., 2011) and as a part of broader interdisciplinary or transdisciplinary learning experiences. Within such educational initiatives, particular attention should also be paid to better supporting first-year students who reported learning significantly less about digital literacies than those in upper years of study. To address continued gaps in meeting students’ needs for digital literacies within the undergraduate curriculum, DL should ideally be embedded within specific disciplinary and professional contexts, as well as overarching curriculum initiatives, such as General Education programs. And while some library initiatives may incorporate aspects of digital literacy as a part of their information literacy (IL) instruction, these are often short, one-time (also known as “one-shot”) sessions, and these time-constraints mean such instruction often cannot provide the in-depth coverage that complex DL topics require. Wider educational and professional development opportunities building DL for faculty educators and other instructional staff across the university, and in turn for students, therefore provide fruitful ground to grow individual and organizational digital literacies.

As an example of such educational development initiatives within our own institutional context, experts from our university’s teaching and learning centre and library partnered to create a workshop for educators on disinformation, “Teaching Students about Fake News: Curriculum Strategies for Navigating Bias and False Content Online.” The goal was building individual and institutional awareness of digital literacies and extending the reach of instructional support for DL across campus (Sharun & Smith, 2020). This workshop helped to forge connections with educators from different disciplines and opened-up opportunities for further one-on-one consultations with faculty about DL in their courses. This example and others like it show possibilities for beneficially integrating disciplinary and transdisciplinary DL approaches, ideally in ways that reach across years of study, to allow for both breadth and depth of coverage. As Cook et. al. (2023) assert in reference to future preparedness, “universities can better support the development of teachers’ digital competence through practical operationalisations that connect technical and pedagogical knowledge” (p. 1), which requires ongoing education and professional development.

### Working toward digital equity

In this study, students identifying as female indicated significantly higher agreement regarding the need for digital literacies to effectively use social media. Canadian studies have shown that young people (aged 18–24) are large users of social media, and that “social media is more popular with women than it is with men” (Gruzd & Mai, 2020, p. 4), though there is variation according to gender for specific social platform use. These gender trends are consistent with reports in other countries (see, for example, the recent Pew Research Center report by Auxier & Anderson, 2021). Our results likewise show gender differences, with a significantly more female students indicating they use social media in their everyday life, though results do not show gender differences for overall use of social media in undergraduate learning.

Notably, not only did female students in this study report higher everyday use of social media, but they also rated their need for digital literacies to effectively use social media as being significantly higher than male students. As Sainz reminds us, it is crucial to reflect upon and respond to the “differences between men and women in the digital uses and competencies acquired” (Vilá, 2021, para. 11), since they are a type of *digital gender gap* presenting a variety of social and economic impacts that put women at risk of being left behind. Further highlighting a need to close this gap (also known as the *digital gender divide*), a report by the Organisation for Economic Co-operation and Development (OECD), which illustrated gender differences in digital literacy, cautioned that a lack of education is one of the key factors that “curtail women and girls’ ability to benefit from the opportunities offered by the digital transformation” (OECD, 2018, p. 5). To address the underlying causes of this gender gap, Davaki’s (2018) recommendations include providing capacity-building opportunities that build literacies through participatory training. Those within and beyond higher education must therefore recognize, as Meyerhoff Nielsen and Erhi Makpor (2021) concluded in their comprehensive review, that “a large global digital gender gap persists….[illustrated in] a disturbing presence of inequalities” (p. 127), issues where the development of digital literacies are of vital importance.

In light of this, developing curriculum in ways that address such gaps across marginalized groups is urgently needed: any future for digital literacy development in the undergraduate curriculum must be done in ways that promote *digital equity*, including and beyond aspects of gender. Working towards digital equity also means meaningfully advancing *digital inclusion* of those who have been marginalized across a range of social, economic, cultural, and political contexts, where an intersectional approach that considers different dimensions of identity, inequality, and marginality brings value (Alper et al., 2016). As an example, the National Digital Inclusion Alliance recommends creating a strong *digital inclusion ecosystem* that includes “multilingual digital literacy and digital skill trainings that meet the community’s needs” (n.d., para. 2), in collaboration with community members. In working to address a community’s diverse needs for digital equity and inclusion in collaboration and partnership with community members (such as students) themselves, embedding an asset-oriented (rather than deficit-based) ethos congruent with an intersectional approach (Alper et al., 2016) can support participatory educational opportunities that aim to build empowerment through digital literacies.

## Limitations and further research

While a random stratified sample was appropriate for the purpose of our study, we note that this sample was intended to be representative of the setting from which it was drawn. We have worked to ensure a robust research design and have used rigourous data collection and analysis strategies, though we also acknowledge common limitations of survey research (e.g., possibilities of nonresponse or measurements errors, response bias, etc.), including some researchers’ critiques that pre-determined survey items may over-simplify complex realities. As such, there is an opportunity for future research on the phenomenon in this study using other research methods and methodologies, including different research designs and sample types, particularly those within qualitative and mixed methods of inquiry. We also recognize the differences between student perceptions of their own learning and other measures (e.g., external metrics, observable changes) of student learning performance, both individually and at an institutional level, and this is an area worthy of additional research. Finally, our research data was collected just months before the COVID-19 pandemic, and while pandemic-related impacts are beyond the scope of our current research, there is an opportunity for future research to consider any changes to and implications for the issues at hand during and after the pandemic.

## Conclusion

Digital literacies, particularly those required for social media, will only continue to increase in importance as digital technologies continue to permeate our lives and our teaching and learning environments. As illustrated in this study’s findings, those in higher education must work to close an observable gap between the high importance that students place on digital literacies required for their learning and their lives as compared to the lack of coverage about these topics students reported receiving in their undergraduate education. There is a clear opportunity to increase coverage of digital literacies within specific courses and across the curriculum in ways that build knowledge and application of essential procedural and technical, cognitive, and sociocultural competencies within and beyond social media spaces. Efforts to foster and promote digital literacies must take into account specific disciplinary and professional contexts, as well as opportunities for interdisciplinary and transdisciplinary learning, such as those within General Education programs. Educational and professional development initiatives with faculty and other instructional staff across the university are also crucial. To fend off the potential for self-enhancement bias, DL initiatives should aim to authentically build self-efficacy, and work to realize opportunities to further digital equity via critical engagement.

When seen as a part of a cyclical lifelong learning process, DL initiatives can aim to proactively meet the needs of students, and of their educators, in continually developing and applying digital literacies. The affordances of social media that students value for collaboration, discussion, information finding and sharing, and practise activities related to their learning can enable interactive, critical engagement and participatory interaction. Our findings highlight the importance of facilitating digital literacies in ways that are carefully and thoughtfully embedded to allow for an adequate breadth and depth of coverage in the curriculum.

## Data Availability

An open dataset is available online (Smith & Storrs, 2023) or by emailing the corresponding author.
